# Under the hood of a moving cell

**DOI:** 10.7554/eLife.81108

**Published:** 2022-07-27

**Authors:** Guillaume Romet-Lemonne

**Affiliations:** 1 Université Paris Cité, CNRS, Institut Jacques Monod Paris France

**Keywords:** actin, cytoskeleton, Arp2/3 complex, load adaptation, force generation, cell mechanics, None

## Abstract

Experiments using purified proteins reveal how the network of filaments that underlie cell movement becomes denser when pushing against a stronger mechanical force.

**Related research article** Li T-D, Bieling P, Weichsel J, Mullins RD, Fletcher DA. 2022. The molecular mechanism of load adaptation by branched actin networks. *eLife* **11**:e73145. doi: 10.7554/eLife.73145.

When a cell moves on a substrate, a network of filaments assembles at its front to generate the force needed to push its membrane forward. This network becomes denser when the cell pushes against a higher opposing load, both in vitro (Bieling et al., 2016) and in migrating cells (Mueller et al., 2017). Yet, how this mechanical adaptation is regulated remains elusive.

Each filament in the network is made up of individual actin molecules, or monomers, that progressively join together to form a tree-like architecture (Svitkina et al., 1997). This process requires three key reactions: adding new actin monomers to the growing ends of filaments (elongation); creating new filaments that branch off existing filaments (branching); and terminating growth by stopping the addition of new monomers (capping). These reactions take place at the membrane against which the filament network is growing and pushing. Actin monomers and capping proteins arrive from the cytoplasm, while the complex that triggers branching is supplied by the membrane (Figure 1).

**Figure 1.**
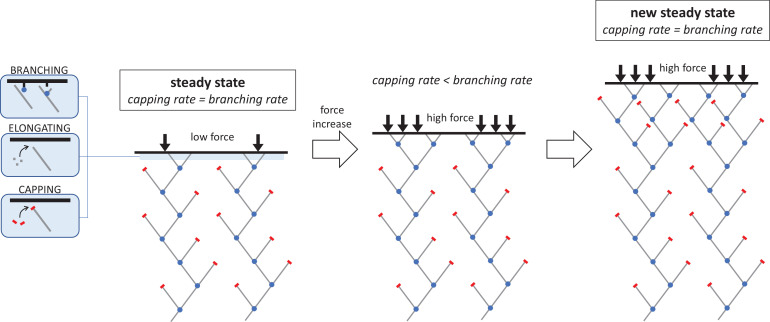
The molecular mechanism responsible for increasing the density of the branched actin network under an increasing mechanical load. The network filaments that push cells forward during migration are made of elongating chains of actin molecules (grey lines) which have protein complexes bound to their ends. This includes the branching complex (blue dots) which forms new actin filaments that branch off the side of existing filaments, and capping proteins (red bars) which stop filament ends from growing. When the system is in a steady state, these two reactions – capping and branching – occur at a similar rate (left). If the opposing force suddenly increases, this causes a sharp drop in capping and elongation. As a result, the rate at which new branched filaments form is higher than the capping rate, leading to more growing ends and a denser network of actin filaments (middle). However, as density increases, the branching rate begins to decline until it matches the capping rate and a new steady state is reached (right). The network in this new, high-force steady state is denser and grows more slowly than in the low-force steady state, but the average filament length remains the same.

Image credit: Guillaume Romet-Lemonne (CC BY 4.0).

Now, in eLife, Tai-De Li, Peter Bieling, Julian Weichsel, Dyche Mullins and Daniel Fletcher report that the network adaptation to increased mechanical pressure is driven by how the capping reaction responds to changes in force (Li et al., 2022). The team (who are based in the United States and Germany) used purified proteins to build a network of branched actin filaments in the laboratory. Different mechanical loads were then applied to the network while elongation, branching and capping were individually monitored over time.

Before the new force is applied, the network is in a low-force steady state where the rate at which new growing ends are formed during branching matches the rate at which filaments are capped. Li et al. show that, when the mechanical load is increased, both the capping and elongation rates decrease exponentially with force via a molecular mechanism called Brownian ratchet. This model proposes that the pushing force reduces the space, which fluctuates due to thermal agitation, between the filament end and the opposing surface. As this space becomes frequently smaller than the molecules targeting the filament ends, they bind less easily and the reaction rate decreases (Peskin et al., 1993). This is the first experimental demonstration of a Brownian ratchet mechanism occurring at actin filament ends, and the first time it has been implicated in the capping reaction.

As the capping rate is now lower, this reaction is occurring slower than branching: more growing ends are being formed than terminated, and the density of actin filaments increases (Figure 1). However, as more new filaments are created, the branching rate starts to decline. A recent study suggests that this might be because the free, growing ends of the filaments disrupt the branching reaction (Funk et al., 2021). To investigate, Li et al. employed a molecular probe they had recently developed (Bieling et al., 2018), and found that this interference mechanism was indeed responsible for the observed drop in branching.

The branching rate then continues to decline until it matches the capping rate. A new higher-force steady state is reached, which allows the now denser network to grow at a constant density. All three reactions occur at lesser rates than in the lower-force steady state, causing the network to expand more slowly. Despite this slow growth rate and increased density, the global architecture of the network remains the same in both states. Because capping proteins and actin monomers are similar in size, the Brownian ratchet effect triggered by the higher opposing force reduces capping and elongation to roughly the same degree. Consequently, the ratio between capping and elongation remains the same, and so does the ratio between branching and elongation. This means that the average filament length between branching points, and between branching points and capped ends, is the same in both steady states.

Although Li et al. have revealed how the assembly of the branched actin network adapts to changes in mechanical force, many questions still remain. For instance, one may wonder how this load-adaptation mechanism is regulated by other proteins, particularly those that control the capping and uncapping of filament ends. In addition, little is known about how the network evolves and is reorganized after it has been assembled (Holz et al., 2022). Indeed, Li et al. report that a significant fraction of the complexes responsible for the branching reaction leave the actin network shortly after integrating into it. Future studies should investigate this intriguing observation as it suggests that unsuspected rearrangements may be rapidly taking place within the assembled network.
